# The NOD/RIP2 Pathway Is Essential for Host Defenses Against *Chlamydophila pneumoniae* Lung Infection

**DOI:** 10.1371/journal.ppat.1000379

**Published:** 2009-04-10

**Authors:** Kenichi Shimada, Shuang Chen, Paul W. Dempsey, Rosalinda Sorrentino, Randa Alsabeh, Anatoly V. Slepenkin, Ellena Peterson, Terence M. Doherty, David Underhill, Timothy R. Crother, Moshe Arditi

**Affiliations:** 1 Division of Pediatrics, Infectious Diseases, and Immunology, Cedars-Sinai Medical Center, David Geffen School of Medicine, University of California Los Angeles, Los Angeles, California, United States of America; 2 Department of Microbiology, Immunology, and Molecular Genetics, University of California Los Angeles, Los Angeles, California, United States of America; 3 Division of Pathology & Laboratory Medicine, Cedars-Sinai Medical Center, David Geffen School of Medicine, University of California Los Angeles, Los Angeles, California, United States of America; 4 Department of Pathology, University of California Irvine, Irvine, California, United States of America; 5 Immunology Research Institute, Cedars-Sinai Medical Center, David Geffen School of Medicine, University of California Los Angeles, Los Angeles, California, United States of America; Stanford University, United States of America

## Abstract

Here we investigated the role of the Nod/Rip2 pathway in host responses to *Chlamydophila pneumoniae*–induced pneumonia in mice. Rip2^−/−^ mice infected with *C. pneumoniae* exhibited impaired iNOS expression and NO production, and delayed neutrophil recruitment to the lungs. Levels of IL-6 and IFN-γ levels as well as KC and MIP-2 levels in bronchoalveolar lavage fluid (BALF) were significantly decreased in Rip2^−/−^ mice compared to wild-type (WT) mice at day 3. Rip2^−/−^ mice showed significant delay in bacterial clearance from the lungs and developed more severe and chronic lung inflammation that continued even on day 35 and led to increased mortality, whereas WT mice cleared the bacterial load, recovered from acute pneumonia, and survived. Both Nod1^−/−^ and Nod2^−/−^ mice also showed delayed bacterial clearance, suggesting that *C. pneumoniae* is recognized by both of these intracellular receptors. Bone marrow chimera experiments demonstrated that Rip2 in BM-derived cells rather than non-hematopoietic stromal cells played a key role in host responses in the lungs and clearance of *C. pneumoniae*. Furthermore, adoptive transfer of WT macrophages intratracheally was able to rescue the bacterial clearance defect in Rip2^−/−^ mice. These results demonstrate that in addition to the TLR/MyD88 pathway, the Nod/Rip2 signaling pathway also plays a significant role in intracellular recognition, innate immune host responses, and ultimately has a decisive impact on clearance of *C. pneumoniae* from the lungs and survival of the infectious challenge.

## Author Summary

*Chlamydophila pneumoniae* (*C. pneumoniae*) is a common intracellular parasite that causes lung infections and contributes to several diseases characterized by chronic inflammation. Toll-like receptors expressed on the cell surface detect *C. pneumoniae* and mount a vigorous defense, but it is not known how the cell defends itself once the pathogen has taken up residence as a parasite. We reasoned that cytosolic pattern recognition receptors called Nods (nucleotide oligomerization domain) that detect microbes that gain entry into the cell might be involved. Using mice genetically deficient in Nod1 and Nod2 or their common downstream adaptor (Rip2), we show that in lung infection, Nod proteins are indeed essential in directing a defense against *C. pneumoniae*. Mice with defective Nod/Rip2-dependent signaling exhibited delayed recruitment of neutrophils, blunted production of pro-inflammatory cytokines and chemokines, and evidence of defective iNOS expression and NO production. These impaired responses led to delayed clearance of bacteria, intense persistent lung inflammation, and increased mortality. By performing bone marrow transplantation experiments and direct transfer of cells into the lungs of mice, we demonstrated that intact Nod-dependent signaling in bone marrow–derived cells was critical in the defense against *C. pneumoniae*. Our results indicate that Nod proteins also play an important role in host defense against *C. pneumoniae*. Coordinated and sequential activation of TLR and Nod signaling pathways may be necessary for an efficient immune response and host defense against *C. pneumoniae*.

## Introduction

*Chlamydophila pneumoniae* is a Gram-negative obligate intracellular pathogen that is widely prevalent [1], causes respiratory tract diseases such as pneumonia, sinusitis, and bronchitis, contributes to acceleration of atherosclerosis [2],[3], and is associated with development of chronic lung diseases such as asthma [4] and other disorders where chronic inflammation is a hallmark feature [5],[6]. *C. pneumoniae* infects various cell types such as epithelial cells, monocytes, macrophages, smooth-muscle cells and endothelial cells, and often resides intracellularly for indefinite periods [7].

*C. pneumoniae* induces a similar lung pathology in humans and rodents [8]. A mouse model of lung infection has been used to study the immunological mechanisms of host defenses. Host immune responses to *C. pneumoniae* proceeds in two stages; 1) an early response requiring IFN-γ to limit the growth of the intracellular bacteria, which plays a central role in the innate control of this infection, and 2) a later adaptive immune response that includes CD4^+^ and CD8^+^ T cells in bacterial clearance and protection [9]–[11]. While the primary immune response is aimed to clear the primary infection from the host and provide protection against reinfection with the same pathogen, generation of tissue injury also occurs and Chlamydial infections often recur or remain persistent and long-term consequences of recurrent or persistent chlamydial infections can be severe [10],[12].

*Chlamydia* is internalized by macrophages as well as by “non-professional” phagocytes, where it survives and replicates. *C. pneumoniae* elicits IFN-γ production in infected bone marrow-derived macrophages [13]. In such cells, IFN-γ synergizes with bacterial products to activate various bactericidal mechanisms, including inducible nitric oxide synthase (iNOS), which leads to production of NO [14],[15], which in turn inhibits chlamydial growth [14],[16],[17].

Molecular motifs derived from *C. pneumoniae* are detected by several pattern recognition receptors, especially Toll-like receptor 2 (TLR2) and TLR4 [18],[19]. TLR4 recognizes chlamydial components such as lipopolysaccharide (LPS) and heat shock protein 60 (cHSP60) [20]–[24], and the intact organism stimulates TLR2 and TLR4-mediated responses [25],[26]. TLR-mediated signaling triggered by *C. pneumoniae*-derived molecules instigates development of an inflammatory innate immune responses and TLR/MyD88 signaling plays an important role in host responses against *C. pneumoniae* infection [18],[19]. Studies from our laboratory indicate that MyD88-null mice with *C. pneumoniae* lung infections are unable to mount a sufficient early inflammatory response against the pathogen [18]. These mice show marked delays in recruiting PMNs, CD8+ and CD4+ T cells to the lungs, and fail to clear the pathogen, but then develop a severe, late-stage, and persistent inflammation characterized by increased IL-1β and IFN-γ production that leads to increased mortality [18]. In contrast, TLR4^−/−^, TLR2^−/−^, and WT mice—all of which can detect *C. pneumoniae* and can signal normally via MyD88, readily recovered from the infection and cleared bacteria normally, indicating that MyD88 is essential to an effective defense, but that TLR2 and TLR4 can both detect the pathogen and are therefore redundant [18],[19].

*C. pneumoniae* has a unique biphasic developmental cycle that occurs within the chlamydial inclusion, a membrane-bound vacuole that is trafficked to the peri-Golgi region, where it avoids fusion with lysosomes and destruction, and are able to replicate intracellularly [27],[28]. Chlamydia-mediated vesicular trafficking events transform the inclusion into a compartment from which chlamydiae can acquire nutrients and interfere with multiple host cell functions [29],[30]. While residing intracellularly, the pathogen presumably is not detected by the cell surface TLR2 and TLR4 receptors; hence, it is unclear how *C. pneumoniae* might be detected and held in check once it has been taken up by the cell. *C. pneumoniae*–infected macrophages can limit bacterial growth by expression of IFN-γ, which in turn is controlled by TLR4/MyD88-dependent pathway. However, since Chlamydia can also induce IFN-γ in the absence of TLR4/MyD88 signaling [31], a potential role for TLR-independent and intracellular recognition receptors, such as the nucleotide oligomerization domain (Nod) proteins, has been suggested [31].

Nod proteins and their adaptor molecule Rip2 also known as RICK or CARDIAK are key components of a family of cytosolic innate immune pattern recognition receptors [32]–[36]. Nod1 and Nod2 recognize molecules in the cytoplasm that originate from bacteria, including peptidoglycan (PGN), a component of bacterial cell walls, and the muramyl dipeptide (MDP) structure found in almost all bacteria [37]. Both Nod1 and Nod2 signal via the serine/threonine Rip2 kinase [34],[38],[39]. Once activated, Rip2 mediates activation of NF-κB and the subsequent production of inflammatory cytokines such as TNF-α and IL-6 [40]–[42]. Although some reports indicate that Nod/Rip2-mediated signaling does not induce IFN-γ [43], other studies show that combined TLR and Nod/Rip2 signaling together can lead to IFN-γ expression [44].

In the present study we show that the Nod/Rip2 signaling pathway is essential to detect intracellular *C. pneumoniae* and direct subsequent innate immune host defenses and bacterial clearance in a mouse model of pneumonia, in addition to the well-established role of the TLR/MyD88 pathway. Rip2^−/−^ mice infected with *C. pneumoniae* displayed an impaired cytokine and chemokine release such as IFN-γ, KC and MIP2, and showed impaired iNOS mRNA expression and NO production, and delayed neutrophil recruitment, which led to delayed bacterial clearance, an intense late-stage and persistent lung inflammation and increased mortality.

## Results

### Rip2^−/−^ mice develop severe and persistent histopathologic lung inflammation following *C. pneumoniae* infection compared to WT mice

**Figure 1:**
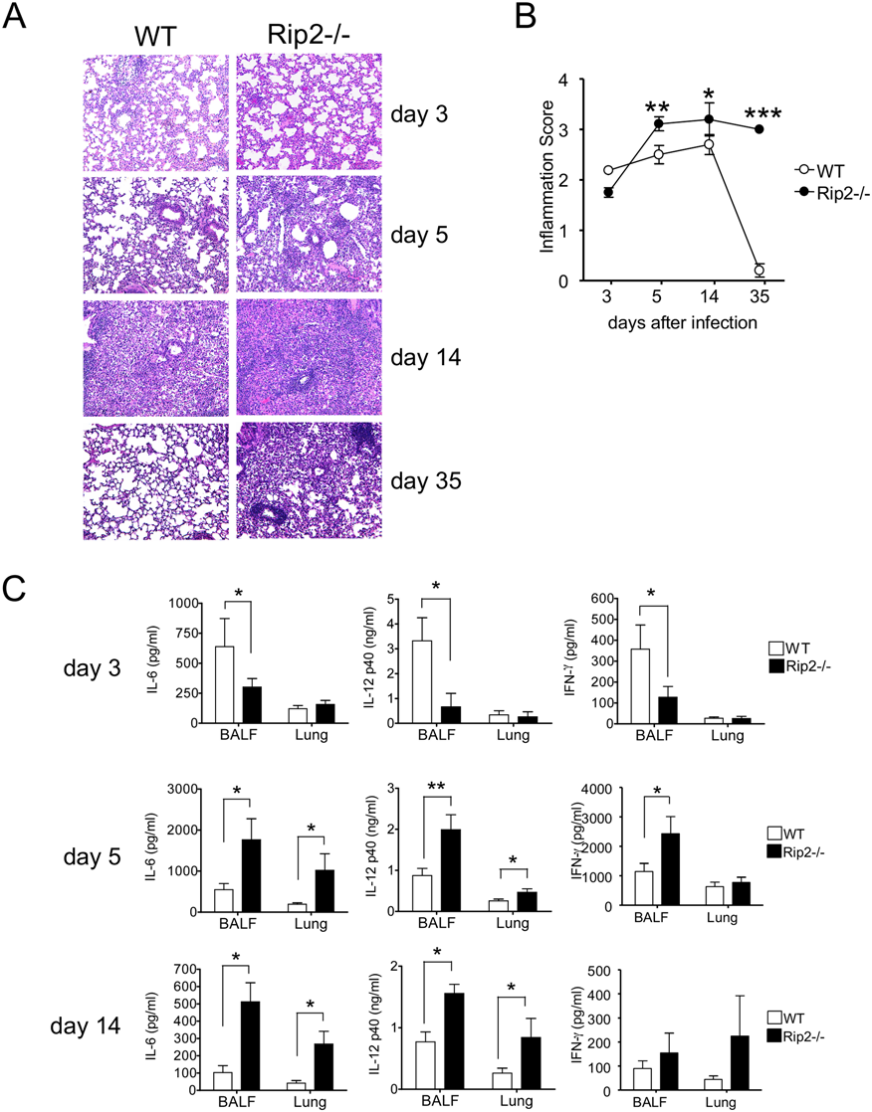
Rip2^−/−^ mice develop a severe and chronic lung inflammation and manifest impaired early cytokine production in response to *C. pneumoniae* infection. WT and Rip2^−/−^ mice were infected with *C. pneumoniae* (1×10^6^ IFU/mouse). Lungs of infected mice were isolated on days 3, 5, 14, and 35 after infection. Lungs were fixed in 10% buffered formalin, sectioned, and stained with H&E to determine the extent of inflammation (A). Histological analysis of inflammation in *C. pneumoniae*–infected lungs of WT and Rip2^−/−^ mice on days 3, 5, 14, and 35 post-infection (B). Statistical significance was determined by Student's *t* test (*p<0.05, **p<0.01, ***p<0.001). At 3, 5, and 14 days post-infection, the levels of IL-6, IL-12p40, and IFN-γ in BALF and lung tissue homogenates were determined (C). Statistical significance was determined by Student's *t* test (*p<0.05, n = 8–14).

Rip2^−/−^ mice and WT controls were infected intratracheally with *C. pneumoniae* (1×10^6^ IFU/mouse) and evaluated for lung inflammation by histopathological analysis. Tissue sections were obtained at 3, 5, 14, and 35 days after infection, fixation and histological staining (H&E) was performed, and sections were graded for degree of inflammation in blinded fashion as detailed in the Materials and Methods Section. As expected, *C. pneumoniae* **–**infected WT mice developed marked lung inflammation as expected by days 5 and 14 and cleared the inflammation and recovered to baseline by day 35 (Figure 1A and 1B). However, Rip2^−/−^ mice developed significantly greater lung inflammation than WT mice by day 5, and day 14, which persisted until the end of the study period at day 35 (Figure 1A and 1B).

### Rip2 deficiency impairs the production of inflammatory cytokines during *C. pneumoniae* infection by day 3

Innate immune responses, and particularly IFN-γ plays an important role in host defense against acute infection and in establishment of persistence of *C. pneumoniae* [10]. We, therefore, determined the production of cytokines such as IL-6, IL-12 p40 and IFN-γ levels in BALF and lung homogenates from infected Rip2^−/−^ and WT mice on days 3, 5 and 14. Concentrations of IL-6, IL-12p40, and IFN-γ were significantly reduced in BALF of Rip2^−/−^ mice at day 3 compared to WT mice (Figure 1C). However, by day 5 and day 14, IL-6, IL-12p40 and IFN-γ concentrations in the BALF and lung homogenates from Rip2^−/−^ mice were significantly increased and exceeded levels in WT mice (Figure 1C). Thus, in addition to increased histopathological inflammation seen in Rip2^−/−^ mice on days 5 and 14 and during the later stages, we observed an initial impaired and delayed kinetics in cytokine production in *C. pneumoniae*–infected Rip2^−/−^ mice on day 3, which was also followed by a significant increased in cytokine production in the lungs on days 5 and 14 (Figure 1C). We next measured IL-6 and IFN-γ levels in the supernatant of infected bone marrow–derived macrophages (BMDM) and whole lung cells *ex-vivo*. *C. pneumoniae* infection-induced cytokine production *ex-vivo* (IL-6, and IFN-γ release) were significantly impaired in both Rip2^−/−^ macrophages and whole lung cells compared to WT macrophages and whole lung cells (Figure S1A–S1D).

### *C. pneumoniae*–infected Rip2^−/−^ mice show delayed bacterial clearance from the lungs and increased mortality

**Figure 2:**
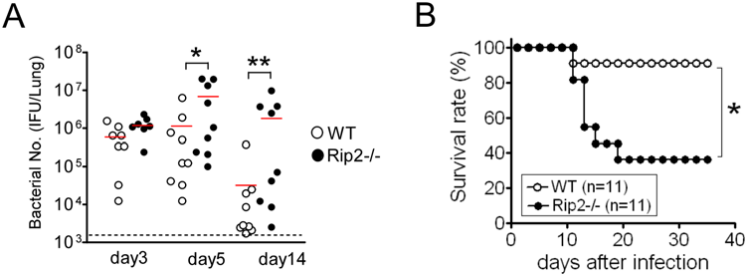
Delayed clearance of *C. pneumoniae* and increased mortality in Rip2^−/−^ mice. WT and Rip2^−/−^ mice were infected with *C. pneumoniae* (1×10^6^ IFU/mouse). At 3, 5, and 14 days post-infection, the bacterial burden in lung tissue homogenates was determined (A). Statistical significance was determined by Student's *t* test (*p<0.05, **p<0.01). Mortality was monitored after infection (B). Statistical significance was determined by Fisher's exact test (*p<0.05, n = 11).

Our data show impaired cytokine production in Rip2^−/−^ mice infected with *C. pneumoniae* early on day 3 following infection but a significant reversal and increase in cytokines and more severe and persistent lung inflammation by day 5 and 14 compared to WT mice (Figure 1). We hypothesized that this more severe and persistent lung inflammation was due to an inability of Rip2^−/−^ mice to clear bacteria, which would then be expected to continue to provoke inflammation and cause the delayed increase in cytokine production. To test this hypothesis, we performed quantitative bacterial cultures in the lungs of mice at days 3, 5, and 14 post-infection. As anticipated, we observed significantly higher numbers of *C. pneumoniae* IFU in the lungs of Rip2^−/−^ mice on days 5 and 14 compared to WT mice (Figure 2A). This could not be explained by higher baseline load of bacteria in Rip2^−/−^ mice, since on day 3, bacterial numbers in lungs were similar between WT and Rip2-deficient mice (Figure 2A). Consistent with the bacterial clearance data, virtually all WT mice survived the infectious challenge, while Rip2^−/−^ mice had significantly increased mortality, and less than half the Rip2^−/−^ mice survived until the end of the experiment at day 35 (Figure 2B). Furthermore, the lungs from the Rip2^−/−^ mice that succumbed to infection harbored an abundance of *C. pneumoniae* (data not shown), while those who survived cleared the bacteria but still manifested chronic lung inflammation at day 35 (Figure 1B). Collectively then, these data indicate that: 1) Rip2 importantly contributes to clearance of *C. pneumoniae* from the lungs; and 2) in the absence of Rip2, severe lung inflammation occurs and persists, but fails to effectively combat the infection.

### Rip2 deficiency impairs early neutrophil recruitment and chemokine expression in the lungs during *C. pneumoniae* infection

**Figure 3:**
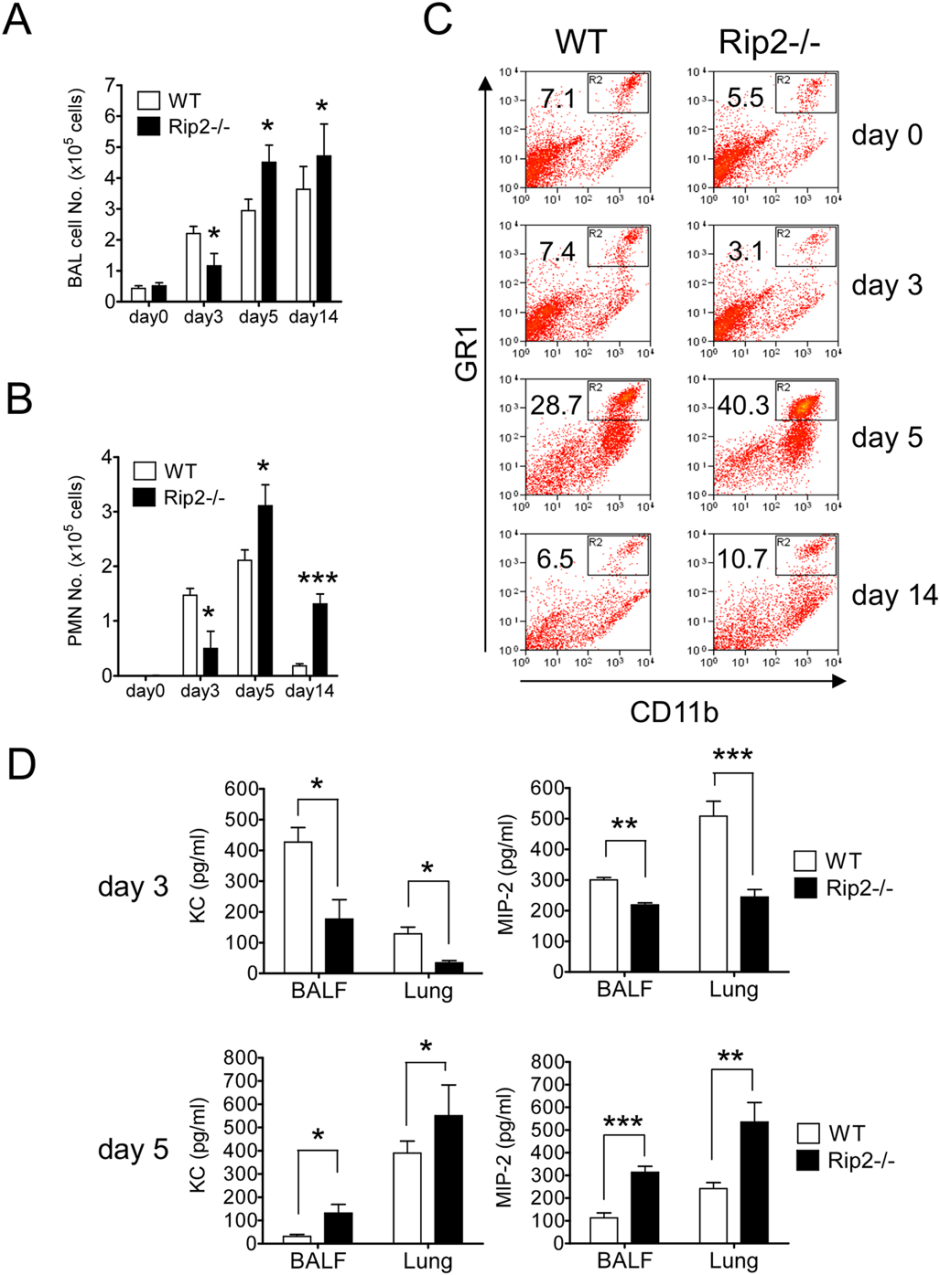
Rip2 deficiency impairs early neutrophil recruitment and chemokine expression in the lungs during *C. pneumoniae* infection. *C. pneumoniae* was inoculated intratracheally into Rip2^−/−^ and WT mice (1×10^6^ IFU/mouse). BALF was collected from WT and Rip2^−/−^ mice on days 0, 3, 5, and 14 post-infection. Total BAL cells (A) and PMN (B) were counted. Statistical significance was determined by Student's *t* test (*p<0.05, ***p<0.001, n = 10∼14). Lungs were removed, and digested with collagenase and DNase I. Cells were stained with PE-anti-GR1 mAB and PECy5-anti-CD11b mAb and analyzed by FACScan (C). The percentages of gated positive cells are indicated. Data shown are representative of four independent experiments. The levels of KC and MIP-2 in BALF and lung tissue homogenates on days 3 and 5 post-infection were determined (D). Statistical significance was determined by Student's *t* test (*p<0.05, **p<0.01, ***p<0.001).

Polymorphonuclear neutrophils (PMN) are crucial for innate host defense against bacteria and fungi. We previously reported that MyD88^−/−^ mice infected with *C. pneumoniae* fails to recruit PMN into the lungs during early and late stages of the infection [18]. To investigate the PMN recruitment in Rip2-deficient mice, we infected Rip2^−/−^ and WT mice with *C. pneumoniae* intratracheally, and compared total cells and PMN in BALF on day 3 and 5 following infection. Both PMN and total BALF cells in Rip2^−/−^ mice were significantly lower compared to WT mice on day 3 following infection (Figure 3A and 3B). However, by days 5 and day 14 post-infection, PMN as well as total BALF cell counts in Rip2^−/−^ mice increased markedly, and were significantly higher than in WT mice (Figure 3A and 3B). Assessment of neutrophil recruitment to the lung by flow cytometric analysis demonstrated similar results (Figure 3C). The percentage of neutrophils (defined by Gr1+ CD11b+ cells) in the lungs of Rip2^−/−^ mice were reduced on day 3 of infection, but increased thereafter, and by days 5 and 14, significantly exceeded the neutrophil percentage of lung cells in WT mice (Figure 3C). We next sought to examine whether the chemokines associated with neutrophil recruitment in the lungs were also affected in the Rip2-deficient mice. Rip2^−/−^ mice showed significantly lower concentrations of KC and MIP-2 in both BALF and lung homogenates compared with WT mice on day 3 after infection (Figure 3D). However, both KC and MIP-2 levels in BALF and lung homogenates significantly increased in Rip2^−/−^ mice compared to WT mice by day 5 (Figure 3D). Collectively, these data indicate that Rip2 plays an important role in early cytokine and chemokine production and neutrophil recruitment to the lungs during the initial days after *C. pneumoniae* infection, and Rip2-deficiency leads to delayed bacterial clearance, which is followed by an exaggerated secondary response consisting of increased cytokine and chemokine expression, PMN recruitment, prolonged, severe histopathological inflammation in the lungs, and increased mortality.

### *C. pneumoniae* is predominantly localized in lung macrophages and PMNs in Rip2-deficient mice

**Figure 4:**
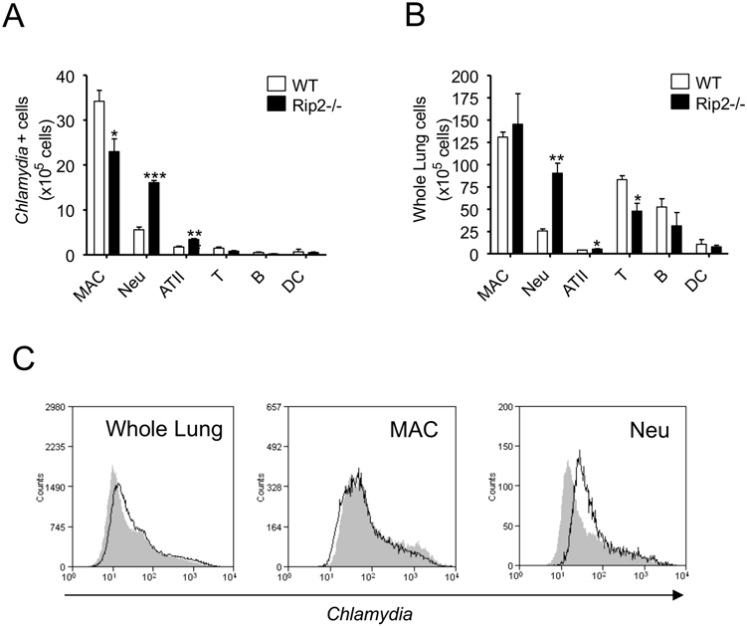
Identification of cell types in the lung harboring *C. pneumoniae*. Single cell suspensions of infected lungs (day 5) were stained with FITC-anti-*Chlamydia* mAb and analyzed by flow cytometry to determine which cell types contained *C. pneumoniae* (A). Composition of lung leukocytes in infected mice (day 5) (B). Statistical significance was determined by Student's *t* test (*p<0.05, **p<0.01). Representative histograms were shown to analyze relative bacterial number in macrophages (MAC) and neutrophils (Neu) between WT (thin line histogram) and Rip2^−/−^ (gray-filled histogram) (C).

Alveolar epithelial cells are the main cells infected in lung infection model [45], but *C. pneumoniae* also infects different cell types including macrophages, dendritic cells, endothelial cells, and PMNs [7]. To determine which cells in the lungs are infected by *C. pneumoniae*, we analyzed infected cell profiles by flow cytometry. *C. pneumoniae* was predominantly found in macrophages and neutrophils, but also in alveolar epithelial cells in the infected lungs (Figure 4A and 4B). Interestingly, in Rip2^−/−^ mice, the number of neutrophils that contained *C. pneumoniae* was significantly increased compared to that in WT mice (Figure 4A). To address whether more bacteria are in Rip2^−/−^ macrophages and neutrophils, we analyzed mean fluorescence intensity (MFI) per cells, which corresponds to relative bacterial number (Figure 4C). We observed a shifted histogram in Rip2^−/−^ neutrophils. These data revealed that neutrophils are likely the main site of Chlamydial replication in lungs at day 5 after infection in Rip2^−/−^ mice.

### Delayed bacterial clearance in Rip2^−/−^ mice is associated with impaired ability to induce iNOS mRNA expression and NO production

**Figure 5:**
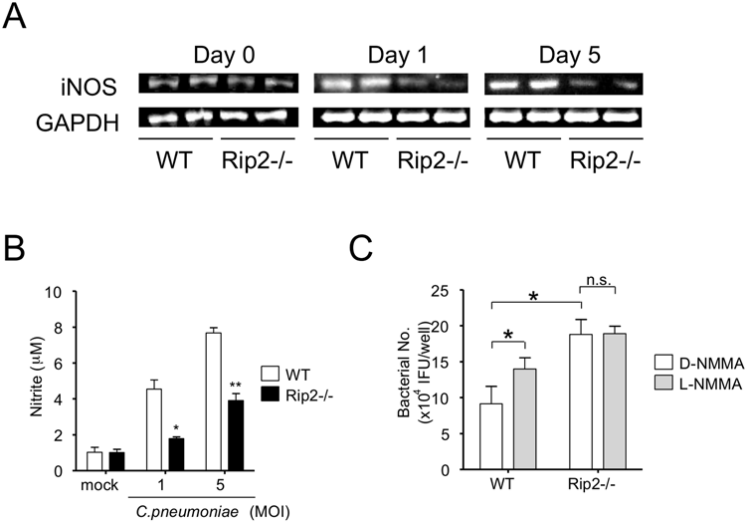
Impaired iNOS mRNA expression and NO production in Rip2^−/−^ mice. Lung tissues of WT and Rip2^−/−^ mice were removed at days 0, 1, and 5 post-infection. iNOS mRNA expression was measured by semi-quantitative RT-PCR (A). Bone marrow–derived macrophages (BMDM) were stimulated with *C. pneumoniae* for 24h. NO production was evaluated by measuring nitrite accumulation in the supernatant using the Griess reaction (B). Statistical significance was determined by Student's *t* test (*p<0.01, **p<0.001, n = 4). *C. pneumoniae* (MOI 5)–infected BMDMs were cultured in the presence of 5mM L-NMMA or D-NMMA for 5 days. Cell lysates were harvested and viable bacteria were quantified by infecting HEp2 cells (C). Statistical significance was determined by one-way ANOVA with Tukey's post-hoc test (*p<0.05, n = 4). Data shown are representative of two independent experiments.

We hypothesized that a bactericidal factor produced by immune effector cells might be responsible for the failure of Rip2^−/−^ mice to clear bacteria. NO produced after cell activation by IFN-γ is important for killing or inhibiting growth of microorganisms [46]. Both IFN-γ and iNOS play major roles in host resistance to chlamydial infection [10]. We therefore assessed the levels of iNOS in the lungs following *C. pneumoniae* infection. Rip2^−/−^ mice demonstrated significantly impaired iNOS mRNA expression compared to WT mice from day 0 until day 5 in total lung cells examined *ex vivo* (Figure 5A). In addition, bone marrow-derived macrophages obtained from Rip2^−/−^ mice, showed significantly diminished NO production following *in vitro* infection with *C. pneumoniae* compared to WT macrophages (Figure 5B). These results suggest that NO production plays a role in killing and clearance of *C. pneumoniae*, and also suggest that Rip2 signaling contributes to NO production in response to *C. pneumoniae* infection. Consistent with this interpretation, *C. pneumoniae* growth was significantly increased in WT macrophages in the presence of an iNOS inhibitor (L-NMMA) compared to control cells treated with an inactive form of the inhibitor (D-NMMA) (Figure 5C). In contrast, *C. pneumoniae* growth was not affected by treatment with the iNOS inhibitor in Rip2^−/−^ macrophages (Figure 5C). Collectively, these results suggest that Rip2-deficient mice have impaired iNOS expression and NO production in response to *C. pneumoniae* infection, which likely contribute to the host immune response defect and delayed bacterial clearance from the lungs of Rip2^−/−^ mice.

### Both Nod1 and Nod2 recognize intracellular *C. pneumoniae* in macrophages and play an essential role in bacterial clearance *in vivo*

**Figure 6:**
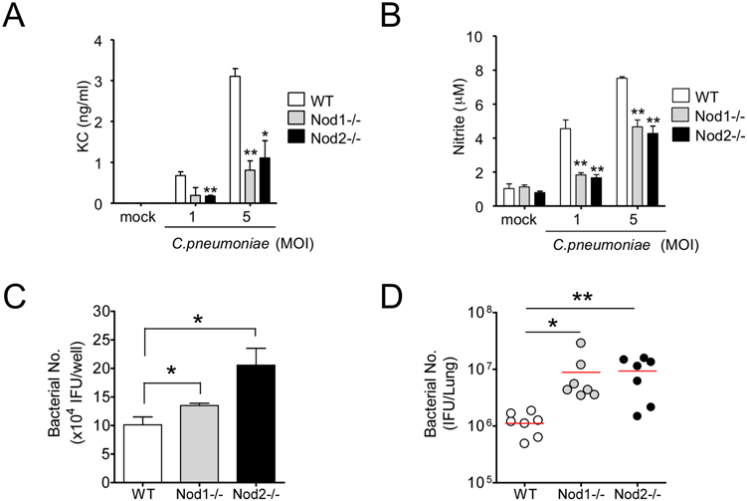
Chemokine and NO production in Nod1^−/−^ and Nod2^−/−^ BMDM and quantitative bacterial counts in lungs of Nod1^−/−^ and Nod2^−/−^ mice following infection with *C. pneumoniae*. WT, Nod1^−/−^, and Nod2^−/−^ BMDMs were infected with *C. pneumoniae* for 24 hours, and KC (A) and NO (B) concentrations in the supernatant were determined. Data shown are representative of three independent experiments. Statistical significance was determined by Student's *t* test (*p<0.05, **p<0.01, n = 3–4). *C. pneumoniae* (MOI 5)–infected BMDM were cultured for 5 days. Cell lysates were harvested and viable bacteria were quantified by infecting HEp2 cells (C). Data shown are representative of two independent experiments. Statistical significance was determined by Student's *t* test (*p<0.05, n = 4). Nod1^−/−^ and Nod2^−/−^ mice were infected with *C. pneumoniae* (1×10*^6^* IFU). After 5 days, lungs were collected and the quantitative bacterial counts in lung tissue homogenates were determined (D). Statistical significance was determined by Student's *t* test (*p<0.05, **p<0.01, n = 7).

Our results thus far indicate that the Rip2^−/−^ mice display an impaired host defenses, delayed bacterial clearance, and increased mortality following *C. pneumoniae* lung infection. Since Rip2 is utilized by both Nod1 and Nod2, we next wished to determine the role of these upstream receptors in *C. pneumoniae* infection. Nod1 was shown to play a role in *C. pneumoniae*-mediated activation of human endothelial cells *in vitro* [47], But it is unclear which Nod receptors detect *C. pneumoniae* in macrophages and during *in vivo* infection. Nod1 is ubiquitously expressed in mammalian cells, but the expression of Nod2 is mainly restricted to primary antigen-presenting cells and epithelial cells, and Nod2 is not expressed in endothelial cells [48]. Furthermore, our data (Figure 4C) indicates that *C. pneumoniae* mainly replicates in macrophages and neutrophils. To determine which Nod receptor recognizes intracellular *C. pneumoniae* in macrophages, we infected Nod1^−/−^ or Nod2^−/−^ BMDM with live *C. pneumoniae* and measured KC and NO levels in the supernatant. Nod1^−/−^ and Nod2^−/−^ macrophages produced significantly diminished KC and NO (Figure 6A and 6B). Consistent with this decreased NO production, bacterial viability was significantly higher in both Nod1^−/−^ and Nod2^−/−^ macrophages *in vitro* (Figure 6C). To determine if greater bacterial viability in the absence of Nod1 and Nod2 also occurred *in vivo*, we infected Nod1^−/−^ and Nod2^−/−^ mice with *C. pneumoniae* and examined bacterial clearance in the lungs. In agreement with the *in vitro* data, both Nod1^−/−^ and Nod2^−/−^ mice displayed delayed pulmonary bacterial clearance compared to WT controls, as reflected by significantly higher bacterial counts in Nod1^−/−^ and Nod2^−/−^ mice at 5 days post-infection (Figure 6D). These results are consistent with the conclusion that intracellular *C. pneumoniae* is recognized by both Nod1 and Nod2 in macrophages, and that signaling emanating from both Nod1 and Nod2 significantly contributes in host defenses against *C. pneumoniae* lung infection, at least in part by regulating production of NO and inflammatory cytokines and chemokines such as IL-12 p40, IFN-γ, KC and MIP2.

### Rip2 in bone marrow–derived cells is important for bacterial clearance of *C. pneumoniae* from the lung

**Figure 7:**
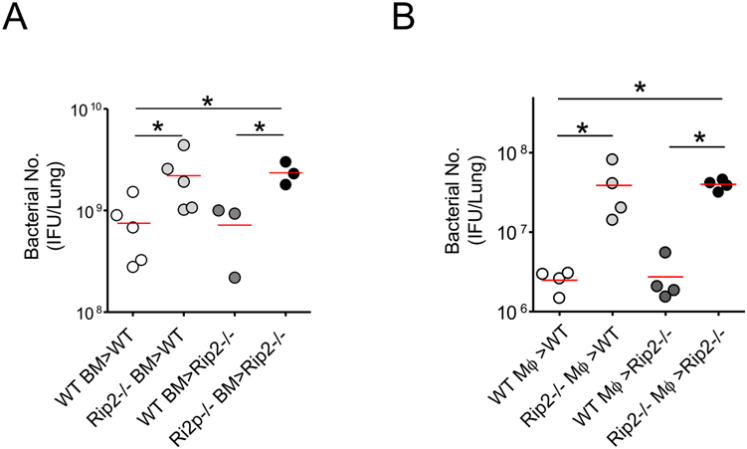
Rip2 in BM-derived cells contributes to bacterial clearance from the lungs. BM chimeric mice were created by lethally irradiating the recipient mice, then performing adoptive transfer and reconstitution with donor BM obtained form WT or Rip2^−/−^ donor mice. After 7 weeks, BM-reconstituted mice were infected with *C. pneumoniae* (1×10^6^ IFU). Lungs were collected on day 5 post-infection. The bacterial burden in lung tissue homogenates was determined (A). BMDMs were grown and isolated from WT or Rip2^−/−^ mice and adoptively transferred intratracheally into the airways of WT mice or Rip2^−/−^ mice (5×10^5^/mouse) together with *C. pneumoniae* (1×10^6^ IFU). BMDMs were first incubated with *C. pneumoniae in vitro* for 30 minutes prior to intratracheal administration. After 5 days, lungs were collected and the bacterial burden in lung tissue homogenates was determined (B). Statistical significance was determined by one-way ANOVA with Tukey's post-hoc test (*p<0.05, n = 3–5).

Based upon data in the previous section that showed involvement of macrophages and PMNs in the lungs, we hypothesized that the Nod/Rip2 signaling pathway in bone marrow (BM)-derived cells rather than non-hematopoietic stromal cells was primarily responsible for innate immune host responses and clearance of *C. pneumoniae* from the lungs. To test this notion, we generated chimeric mice using donor marrow from WT or Rip2^−/−^ mice (Figure S2), then infected the mice intratracheally with *C. pneumoniae*. Five days after infection, lungs were harvested and quantitative bacterial counts were determined. WT recipient mice that were transplanted with Rip2^−/−^ BM displayed significantly higher bacterial load in the lungs compared to control WT mice transplanted with WT BM (Figure 7A). Conversely, Rip2^−/−^ recipient mice that had been transplanted with WT BM displayed lower bacterial counts than control Rip2^−/−^ mice that had received Rip2^−/−^ BM (Figure 7A). In all chimeric mice, we observed generally higher bacterial titers observed in the lungs, most likely due to inherently increased susceptibility secondary to the irradiation procedure itself, as has been previously reported by other investigators [49]. These data indicate that Rip2 in BM-derived cells primarily mediates host defenses against pulmonary *C. pneumoniae* infection. However, it is possible that airway epithelial cells also contribute and play a role in *C. pneumoniae* detection in the lung. In order to further elucidate the primary role of macrophages in *C. pneumoniae* infection, we performed intratracheal adoptive transfer of WT or Rip2^−/−^ BMDM, simultaneously with *C. pneumoniae* infection (i.e. macrophages mixed with bacteria), and then determined the effect on local bacterial replication in lungs of WT or Rip2^−/−^ mice. As anticipated, adoptive transfer of Rip2^−/−^ macrophages plus *C. pneumoniae* into WT mice resulted in significantly higher bacterial counts compared to WT macrophages plus *C. pneumoniae* transferred into WT mice (Figure 7B). However, WT macrophages plus *C. pneumoniae* adoptively transferred into Rip2^−/−^ mice rescued the Rip2 phenotype, i.e. restored bacterial clearance (Figure 7B) and neutrophil recruitment in the lungs (Figure S3), indistinguishable from control WT mice that received WT macrophages plus *C. pneumoniae.* We did not observe a defect in phagocytosis in Rip2^−/−^ macrophages using labeled *C. pneumoniae* (Figure S4) [50]. Taken together, these findings indicate that the Nod/Rip2 signaling pathway in BM-derived cells play a dominant role in bacterial clearance of *C. pneumoniae* from the lungs.

## Discussion

Here we show that cytoplasmic Nod proteins are importantly involved in generating innate immune defenses against intracellular *C. pneumoniae*. We found that deletion of Rip2, that is essential for both Nod1- and Nod2-mediated signaling, delays neutrophil recruitment to the lungs, and suppresses expression of chemokines and cytokines that are essential to generate an effective host defense. Although an inflammatory innate immune response was delayed, by day 5 Rip2^−/−^ mice infected with *C. pneumoniae* developed a more severe inflammation that persisted longer compared to WT mice, but nevertheless failed to clear the pathogen, and most infected Rip2^−/−^ mice ultimately succumbed to the infection. The inability of Rip2^−/−^ mice to eradicate the pathogen despite robust inflammation was associated with delayed kinetics of IL-12p40 and IFN-γ production and suppressed iNOS expression and NO production, all of which are critically important elements in the innate immune armamentarium [9],[11]. Experiments with bone marrow-derived macrophages demonstrated that both Nod1 and Nod2 were involved in sensing intracellular *C. pneumoniae*. Results from experiments with bone marrow chimeric mice confirmed that cells derived from hematopoietic lineages rather than resident stromal cells were essential in Nod/Rip2-mediated defenses against *C. pneumoniae*. This conclusion was corroborated by adoptive transfer of WT or Rip2^−/−^ macrophages directly into the airways of infected mice. Collectively, our data demonstrate that proper functioning of the Nod/Rip2 cytoplasmic innate immune detection system critically determines whether the host can effectively resist and eradicate an infectious challenge to the lungs from *C. pneumoniae*.

Our results underscore previous reports that increasingly emphasize the central role Nod/Rip2 signaling can play in defending the host against intracellular invasion. Nod proteins mediate host defense against a variety of both Gram negative and Gram positive bacteria. For example, Nod2 senses the PGN produced by *Staphylococcus aureus*, and Rip2 limits *S. aureus* growth in macrophages [51]. Also, Nod2 detects the MDP structure found in almost all bacteria [37]. Nod1 is required for IkK and NF-κB activation in human colon epithelial cells infected with *E. coli* [52], participates in KC induction and impacts bacterial viability to *Pseudomonas aeruginosa* in mouse embryonic fibroblasts [53]. Nod2 triggers cytokine production by DCs in response to live *M. tuberculosis,* but is not essential to control *M. tuberculosis* airway infection [54]. While Rip2 and Nod1 deficiency increases susceptibility to *Listeria monocytogenes* [32] and *Helicobacter pylori* [55] respectively, neither Nod1 nor Rip2 deficiency had any significant effect on *Chlamydia muridarum* vaginal infection [56]. Hence, available data indicates that Nod1 and Nod2 selectively interact with specific pathogens and can play a critical role in host defenses, highlighted by increased susceptibility to specific pathogens in mice lacking these intracellular receptors. Accordingly, our data now indicate that Nod/Rip2 signaling is also essential in successfully combating *C. pneumoniae* lung infection. Indeed, in the absence of Nod/Rip2 signaling, lung infection with *C. pneumoniae* proved fatal to the majority of mice.

Clearly, one reason for the increased susceptibility of the Rip2^−/−^ mice to *C. pneumoniae* lung infection may be the inability of Rip2^−/−^ mice to rapidly recruit neutrophils to the site of infection. Delayed neutrophil recruitment in Rip2^−/−^ mice appears closely linked to lack of sufficient chemokine and cytokine expression compared to infected WT mice. However, by days 5 and 14 post-infection, the percentage of neutrophils in the lungs of Rip2^−/−^ mice increased and significantly exceeded those in WT mice. Evidently, this delayed early response to the infectious challenge allows the bacteria to gain the upper hand, but despite the significant increase in PMN numbers on days 5 and 14 post-infection, the Rip2^−/−^ mice showed delayed bacterial clearance from the lungs and increased mortality. Interestingly, in Rip2^−/−^ mice, the number of neutrophils in the lungs that contained *C. pneumoniae* was significantly increased compared to that in WT mice. Several obligate intracellular microbial pathogens develop mechanisms to evade destruction upon ingestion by PMN [57],[58]. Indeed, *C. pneumoniae* can infect and replicate in PMNs and these cells in turn can enhance replication and Chlamydial burden during infection [59].

However, the main reason Rip2^−/−^ mice cannot clear the infection and most often succumb to the disease appears to be more closely tied to our observation that expression of IL-12p40, IFN-γ, iNOS and NO are suppressed in the absence of Rip2^−/−^. These data are consistent with previous reports, which similarly indicate that IL-12p40 [9], IFN-γ [9],[60] and iNOS [11],[61] are essential for effective host resistance against *C. pneumoniae* infection. Several studies showed that IFN-γ and IL-12 both play an important role in the innate control of *C. pneumoniae* infection [9],[11] IL-12 participates in resistance to *C. pneumoniae,* likely by enhancing IFN-γ mRNA [9]. In turn, IFN-γ produced by innate cells increases iNOS expression and NO release and controls the intracellular growth of *C. pneumoniae* [14],[16],[17]. Increased susceptibility of IFN-γR^−/−^ mice is associated with diminished levels of iNOS mRNA accumulation in lungs, and iNOS^−/−^ mice also show higher sensitivity to *C. pneumoniae* infection [11]. However, IFN-γR^−/−^ mice shows even greater sensitivity to *C. pneumoniae* infection compare to iNOS^−/−^ mice, suggesting the presence of both iNOS-dependent and -independent IFN-γ-mediated effector mechanisms [11].

IFN-γ can be produced by cells from both the innate and acquired immune system. The susceptibility of IFN-γR^−/−^ mice largely exceeds that of RAG-1^−/−^ mice, suggesting an important role for non-T cell-mediated IFN-γ-producing cells in the host resistance against *C. pneumoniae* infection [11]. Several studies show that, besides NK and T cells, myeloid cells such as macrophages, DCs and neutrophils can also express IFN-γ [62].

Previous studies have shown that MDP induced NO production in macrophages [63],[64]. Several other reports also suggest a link between Nod and NO production [64]. Our results indicating that Nod/Rip2 signaling stimulates iNOS expression and NO production suggest that one of the reasons why *C. pneumoniae* lung infection proves lethal to most Rip2^−/−^ mice is because they fail to generate an effective NO-mediated defense by immune effector cells, and thus cannot eradicate the pathogen.

In addition to innate immunity, adaptive immune responses may also directly or indirectly diminish the levels of IFN-γ and IL-12 mRNA early after infection and thus may alter the quality of the protective host immune responses. A protective role for CD8 T cells is shown by the higher sensitivity and enhances severity of infection in CD8^−/−^ mice [11]. In MyD88^−/−^ mice we observed a delay in recruitment of CD4 and CD8 T cells into the lungs [18], while in the current study with Rip2^−/−^ mice we observed primarily a delay CD4 T cell recruitment initially followed by significant increases in the presence of both CD4 and CD8 T cells later on day14 (Figure S5).

Our data with bone marrow chimeric mice clearly demonstrate that the cells responsible for Nod/Rip2-dependent defense against *C. pneumoniae* are hematopoietic in origin, and are not resident stromal cells. Additionally, adoptive transfer of WT macrophages was able to rescue the bacterial clearance defect in Rip2^−/−^ mice. While our data do not completely rule out a potential role for other cell types during *C. pneumoniae* infection, the bactericidal effects of the Nod/Rip2 pathway appear to be predominantly of bone marrow origin with macrophages playing the largest role.

*C. pneumoniae*-induced IL-12p40 production *in vivo* involves both MyD88-dependent and MyD88-independent pathways [59], suggesting a TLR-independent, but Nod-dependent mechanism of recognition and activation. Indeed, recent studies suggest that in endothelial cells, Nod1 plays an important role in triggering *C. pneumoniae*-mediated inflammatory responses [47], and that Nod1 is involved in NF-κB activation by Chlamydia in epithelial cell lines [56]. Furthermore, a recent study by Buchholz *et. al*. concluded that *C. trachomatis* induced IL-8 responses are dependent on Nod1 and Rip2 signaling in Hela cells [65]. Our *in vivo* data showing that *C. pneumoniae* induced chemokine production in the lungs depends on Rip2 signaling is consistent with the *in vitro* observations by Buchholz *et. al.* These findings suggest that PGN fragments are synthesized by chlamydiae and are recognized by the host innate immune system. The genome sequence revealed that Chlamydophila is actually equipped with a full complement of PGN synthesis genes [66]. Chlamydia is sensitive to antibiotics like penicillin that inhibit PGN synthesis [67],[68], but clear-cut biochemical evidence for the synthesis of PGN in chlamydiae is missing [69],[70]. A recent study revealed the biochemical capacity of *C. trachomatis* to synthesize m-DAP and that the m-DAP synthesis genes are expressed as early as 8h after infection [71]. This paradox, known as the ‘chlamydial anomaly’ is still being debated in the light of genomic information [72]. However, prior studies and our current data suggest that chlamydial PGN released by bacteria must make their way across the inclusion membrane into the cytosol. One potential mechanism by which this could occur is through the proposed type III secretion system [73]. A similar mechanism of type IV secretion has been proposed for Nod1 signaling in *H. pylori* infection [55]. While the exact ligand(s) of *C. pneumoniae* detected by the Nod are yet to be identified, our data clearly indicate that both Nod1 and Nod2 recognize *C. pneumoniae* and play an essential role in host defenses against this microorganism. Our data differ from those obtained in experimental infections with *C. trachomatis* or *C. muridarum* genital tract infection, where Nod1 deficiency had no significant effect on the efficiency of infection, or pathology in vaginally infected mice, while Rip2-deficient mice had only slightly increased bacterial load and delayed bacterial clearance and mildly increased oviduct inflammation [56]. Such differences are not surprising, as the two organisms display only 5 and 10% homology at the DNA and protein levels, respectively, as also reflected in the different pathobiologies they cause [74].

In summary, we demonstrate that the Nod cytosolic pattern recognition receptors are essential for mounting an adequate defense against *C. pneumoniae*, that Nod stimulate chemokine and cytokine production and neutrophil recruitment in the early phase of infection, and that the cells responsible for the effects of Nod are bone marrow-derived cells, not stromal cells. Furthermore, we show that Nods stimulate IL12-p40, IFN-γ, iNOS and NO expression, and that these factors are key for surviving the infectious challenge. Since the TLR/MyD88 pathway is also critically involved in detecting and eradicating *C. pneumoniae*, our data highlight an emerging theme in host defenses: that divergent pattern recognition receptors that are seemingly unrelated and expressed in distinct compartments can nevertheless direct cooperative responses that successfully combat invasion by common pathogens such as *C. pneumoniae*. Coordinated and sequential activation of TLR and Nod signaling pathways may be necessary for efficient immune responses and host defenses against *C. pneumoniae*. While TLRs might be important for initial activation upon Chlamydophila contact, it is likely that Nod proteins play a role in the sequential and intracellularly triggered prolonged activation of target cells by intracellular Chlamydophila.

## Materials and Methods

### Mice

Rip2^−/−^ mice, backcrossed ten generation to C57BL/6, were kindly provided by Dr. Genhong Cheng (University of California at Los Angeles, Los Angeles, CA, USA). C57BL/6 mice and Nod2^−/−^ mice were purchased from Jackson Laboratory. Nod1^−/−^ mice were kindly provided by Dr. Jeffrey Weiser (University of Pennsylvania, Philadelphia, PA, USA). Mice were maintained under specific pathogen-free conditions, and were used at 8–12 weeks of age. All experiments were done according to Cedars-Sinai Medical Center Institutional Animal Care and Use Committee guidelines.

### *C. pneumoniae* infection

*C. pneumoniae* CM-1 (ATCC, Manassa, VA) was propagated in HEp-2 cells as previously described [18]. HEp-2 cells and *C. pneumoniae* stocks were determined to be free of *Mycoplasma* contamination by PCR. Mice were intratracheally infected with *C. pneumoniae* by inoculating 100 µl of PBS containing 1×10^6^ IFU of the microorganism. Bronchoalveolar lavage fluid (BALF) was collected with 0.5 ml of PBS containing 2mM EDTA. The lavage fluid was centrifuged, and the supernatant was used for chemokine and cytokine measurements. The pellet placed on glass slides, and stained by modified Wright-Giemsa staining (Diff-Quick; Fisher Scientific, Pittsburgh, PA, USA) to determine leukocyte subtypes based on their cellular and nuclear morphology. Lungs were homogenized with 1ml of sucrose-phosphate-glutamate medium and stored at −80°C.

### Bacterial quantification

To quantify *C. pneumoniae* progeny, HEp2 cells were inoculated with lung specimens or cell lysates as previously described [75]. Briefly HEp2 cells were infected with diluted lung homogenates or infected cell lysates. Cultures were centrifuged for 1h at 800× g, fed with RPMI1640 in the presence of cycloheximide (1 µg/ml), and incubated for 72h. Thereafter, Cells were washed with PBS, fixed with methanol for 5 min at room temperature and stained with FITC-conjugated *Chlamydia* genus-specific mAb (Pathfinder *Chlamydia* Culture Confirmation System; BIO-RAD, Hercules, CA, USA) according the manufacturer protocol. Inclusion bodies were counted by fluorescence microscopy.

### Histopathological analysis

Lungs were fixed in formalin buffer, paraffin-embedded, and hematoxylin and eosin-stained sections were scored by a trained pathologist blinded to the genotypes as previously described [18]. Briefly, the degree of inflammation was assigned an arbitrary score of 0 (normal = no inflammation), 1 (minimal = perivascular, peribronchial, or patchy interstitial inflammation involving less than 10% of lung volume), 2 (mild = perivascular, peribronchial, or patchy interstitial inflammation involving 10–20% of lung volume), 3 (moderate = perivascular, peribronchial, patchy interstitial, or diffuse inflammation involving 20–50% of lung volume), and 4 (severe = diffuse inflammation involving more than 50% of lung volume).

### Detection of chemokines and cytokines

The chemokine and cytokine concentrations in the BALF, lung homogenates or culture supernatant were determined using by Duoset Mouse KC, MIP-2 (R&D systems, Minneapolis, MN, USA), OptiEIA Mouse IL-6 ELISA Set (BD Biosciences, San Jose, CA, USA) and Mouse IFN-γ ELISA, Mouse IL-12 p40 ELISA (eBioscience, San Diego, CA, USA). The assays were performed as described manufacturer protocol.

### Flow cytometric analysis

The lymphocytic makeup in the lungs after infection were analyzed by flow cytometry of lung homogenates. Briefly, lymphocytes were isolated by digesting the lung tissue at 37°C for 1h with HANKS' containing 100 µg/ml Blenzyme (Roche Diagnostics, Indianapolis, IN, USA) and 50 units/ml DNase I (Roche Diagnostics) and filtering through a 70 µm cell strainer (BD Biosciences). Erythrocytes were depleted by lysis buffer before staining. Isolated single cells were stained with following specific mAbs; CD16/32 (clone 93), Gr1 (clone RB6-8C5), CD11b (clone M1/70), F4/80 (clone BM8), CD11c (clone HL3), CD45 (clone 30-F11), CD4 (clone RM4-5) and CD8 (clone 53-6.7) were purchased from eBioscience as direct conjugates to FITC, PE or PECy5. Anti SP-C polyclonal Ab and PEcy5-conjugated donkey anti-Goat IgG F(ab') were used for Alveolar type II epithelial (ATII) cell staining (Santa Cruz Biotechnology, Santa Cruz, CA, USA). Cells were identified based on expression of following antigens: pulmonary macrophages (F4/80+ and CD11c+), DC (F4/80− and CD11c+), Neutrophils (Gr1+ and CD11b+), ATII cells (SP-C+, CD45- and CD16/32-), T cells (CD3+), B cells (CD19+). For intracellular Chlamydophila staining, cells were permeabilized using Cytofix/Cytoperm kit (BD Biosciences) and stained with FITC-conjugated anti-*Chlamydia* LPS mAb (Accurate Chemical and Scientific Corporation, Westbury, NY, USA). Flow cytometric analysis was performed by FACScan flow cytometer (BD Biosciences) and the data was analyzed by Summit (Dako, Carpinteria, CA, USA).

### RT-PCR

Total RNA was extracted from homogenized lung tissues by RNeasey mini kit (QIAGEN, Valencia, CA, USA) following the manufacturer's protocol. Total RNA preparations were subjected to reverse transcriptase-polymerase chain reaction analysis by Total cDNA was generated using the Omniscript cDNA synthesis kit (Qiagen), PCR analysis was performed using specific primers for mouse iNOS (sense: 5′-TGG GAA TGG AGA CTG TCC CAG-3′:antisense: 5′-GGG ATC TGA ATG TGA TGT TTG-3′), 1min at 94°C , 1 min at 58°C and 2 min at 68°C. Amplification of GAPDH served as a control.

### Preparation of bone marrow–derived macrophages (BMDM)

Femora and tibiae of mice were rinsed with cell culture medium. Bone marrow cells were treated with red blood lysis buffer (eBiosciences), cultured in RPMI1640 medium containing 10% FBS and 10 ng/ml M-CSF (R&D system). Medium changed at day 3 and day 6. BMDM were harvested at day 9 and exposed to *C. pneumoniae* by centrifugation at 500× g for 30 min.

### Nitrite assay

Nitrite levels in the culture supernatant were determined using the colorimetric Griess reaction (Sigma, St. Louis, MO, USA). Absorbance was measured with a plate reader at 540 nm. The concentration of NO_2_^−^ was determined from standard curves constructed with serial concentrations of NaNO_2_.

### Generation of BM chimeric mice

Recipient WT (Ly5.1), WT (Ly5.2) and Rip2^−/−^ (Ly5.2) mice were lethally γ-irradiated with 950 rads using a ^137^Cs γ-source and were reconstituted intravenously with 5×10^6^ BM cells derived from respective donors 2–3h later. All mice were placed on Baytril (Bayer HealthCare LLC, Shawnee Mission, KS, USA) for 2 weeks following irradiation. 6–7 weeks after engraftment, mice were tested by FACS analysis with FITC-conjugated Ly5.2 Ab (clone 104, eBiosciences) and PE-conjugated Ly5.1 Ab (clone A20, eBiosciences) staining for chimerism.

### Statistics

Data are reported as mean values±S.D. Statistical significance was evaluated by Student's *t* test. In the case of survival study, Statistical significance was evaluated by Fisher's exact test. For multiple comparison test, Statistical significance was evaluated by one way ANOVA with Tukey's post-hoc test.

## Supporting Information

Figure S1 IL-6 and IFN-γ production in Rip2^−/−^ BMDM and whole lung cells. BMDM (2×105 cells) were stimulated with *C. pneumoniae* (Cpn) for 24 hours. IL-6 (A) and IFN-γ (B) concentration in the supernatant was evaluated by ELISA. Whole lung cells (2×105 cells) were stimulated with *C. pneumonia* (Cpn) for 24 hours. IL-6 (C) and IFN-γ (D) concentration in the supernatant was evaluated by ELISA. Data shown are representative of three independent experiments. Statistical significance was determined by Student's t test (*p<0.05, **p<0.01, n = 3).
(0.32 MB TIF)

Figure S2 Reconstitution of BM chimeras. Recipient mice were lethally irradiated and reconstituted with BM from WT or Rip2^−/−^ donor mice. After 7 weeks, lungs were removed, digested with collagenase and DNase I. Cells were stained with FITC-anti-Ly5.2 mAB and PE-anti-Ly5.1 mAb and analyzed by FACScan.
(0.25 MB TIF)

Figure S3 Neutrophil recruitment after WT BMDMs were adoptively transferred into Rip2^−/−^ mice. BMDMs were grown and isolated from WT or Rip2^−/−^ mice and adoptively transferred intratracheally into the airways of WT mice or Rip2^−/−^ mice (5×105/mouse) together with *C. pneumoniae* (1×106 IFU). BMDMs were first incubated with *C. pneumoniae* in vitro for 30 minutes prior to intratracheal administration. After 3 days, lungs were collected and cells were stained with PE-anti-GR1 mAB and PECy5-anti-CD11b mAb and analyzed by FACScan. The percentages of gated positive cells are indicated. Data shown are representative of two independent experiments.
(0.64 MB TIF)

Figure S4 Phagocytosis of labeled *C. pneumoniae* by BMDM is unaffected by targeted deletion of the gene for Rip2. The bacteria were incubated with DyLigntTM 633 NHS-Ester reagent (Thermo Scientific, Rockford, IL, USA) for 1 hour at RT. FBS was added to stop the reaction, washed with PBS, and centrifuged at 18,000 rpm (60,000× g) for 1 hour. The supernatant was carefully aspirated and the bacterial pellet was resuspended in cell culture medium. BMDMs were exposed to labelled *C. pneumoniae* (solid line histogram) or vehicle control (gray-filled histogram) by centrifugation at 500× g for 30 minutes at 4°C, then incubated for 2 hours at 37°C. MOIs of 10, 20, 40, and 80 were used. Uninternalized bacteria were removed by incubating the cells in Trypsin/EDTA for 10 minutes at 37°C as previously described [75]. The cells were washed and fixed with 2% formalin/PBS, and analyzed by FACS. The mean fluorescence intensity (MFI) and percentage of labeled *C. pneumoniae* internalized cells were indicated. Data are representative of two independent experiments.
(0.50 MB TIF)

Figure S5 Delayed CD4 T cell recruitment to the lungs in Rip2^−/−^ mice. *C. pneumoniae* was inoculated intratracheally into Rip2^−/−^ and WT mice (1×1 06 IFU/mouse). Lungs were removed on days 0, 3, 5, and 14 post-infection, digested with collagenase and DNase I. Cells were stained with FITC-anit-CD4 mAb, PE-anti-CD3 mAB, and PECy5-anti-CD8 mAb, and analyzed by FACScan. The total number of CD4^+^CD3^+^ (A) and CD8^+^CD3^+^ (B) T cells in lungs were counted. Data shown are representative of four independent experiments. Statistical significance was determined by Student's t test (*p<0.05).
(0.15 MB TIF)

## References

[1] Karvonen M, Tuomilehto J, Pitkäniemi J, Naukkarinen A, Saikku P (1994). Chlamydia pneumoniae IgG antibody prevalence in south-western and eastern Finland in 1982 and 1987.. Int J Epidemiol.

[2] Campbell LA, Kuo CC (2004). Chlamydia pneumoniae—an infectious risk factor for atherosclerosis?. Nat Rev Mirobiol.

[3] Gupta S, Leatham EW (1997). The relation between Chlamydia pneumoniae and atherosclerosis.. Heart.

[4] Hansbro PM, Beagley KW, Horvat JC, Gibson PG (2004). Role of atypical bacterial infection of the lung in predisposition/protection of asthma.. Pharmacol Ther.

[5] Belland RJ, Ouellette SP, Gieffers J, Byrne GI (2004). Chlamydia pneumoniae and atherosclerosis.. Cell Microbiol.

[6] Kuo CC, Jackson LA, Campbell LA, Grayston JT (1995). Chlamydia pneumoniae (TWAR).. Clin Microbiol Rev.

[7] Hammerschlag RM (2002). The intracellular life of chlamydiae.. Semin Pediatr Infect Dis.

[8] Yang ZP, Kuo CC, Grayston JT (1993). A mouse model of Chlamydia pneumoniae strain TWAR pneumonitis.. Infect Immun.

[9] Rottenberg ME, Gigliotti Rothfuchs A, Gigliotti D, Ceausu M, Une C (2000). Regulation and role of IFN-gamma in the innate resistance to infection with Chlamydia pneumoniae.. J Immunol.

[10] Rottenberg ME, Gigliotti-Rothfuchs A, Wigzell H (2002). The role of IFN-gamma in the outcome of chlamydial infection.. Curr Opin Immunol.

[11] Rottenberg ME, Gigliotti Rothfuchs AC, Gigliotti D, Svanholm C, Bandholtz L (1999). Role of innate and adaptive immunity in the outcome of primary infection with Chlamydia pneumoniae, as analyzed in genetically modified mice.. J Immunol.

[12] Ward ME (1995). The immunobiology and immunopathology of chlamydial infections.. APMIS.

[13] Rothfuchs AG, Gigliotti D, Palmblad K, Andersson U, Wigzell H (2001). IFN-alpha beta-dependent, IFN-gamma secretion by bone marrow-derived macrophages controls an intracellular bacterial infection.. J Immunol.

[14] Mayer J, Woods ML, Vavrin Z, Hibbs JBJ (1993). Gamma interferon-induced nitric oxide production reduces Chlamydia trachomatis infectivity in McCoy cells.. Infect Immun.

[15] Beatty WL, Byrne GI, Morrison RP (1993). Morphologic and antigenic characterization of interferon gamma-mediated persistent Chlamydia trachomatis infection in vitro.. Proc Natl Acad Sci U S A.

[16] Igietseme JU, Uriri IM, Chow M, Abe E, Rank RG (1997). Inhibition of intracellular multiplication of human strains of Chlamydia trachomatis by nitric oxide.. Biochem Biophys Res Commun.

[17] Igietseme JU, Perry LL, Ananaba GA, Uriri IM, Ojior OO (1998). Chlamydial infection in inducible nitric oxide synthase knockout mice.. Infect Immun.

[18] Naiki Y, Michelsen KS, Schröder NW, Alsabeh R, Slepenkin A (2005). MyD88 is pivotal for the early inflammatory response and subsequent bacterial clearance and survival in a mouse model of Chlamydia pneumoniae pneumonia.. J Biol Chem.

[19] Rodriguez N, Wantia N, Fend F, Dürr S, Wagner H (2006). Differential involvement of TLR2 and TLR4 in host survival during pulmonary infection with Chlamydia pneumoniae.. Eur J Immunol.

[20] Da Costa CU, Wantia N, Kirschning CJ, Busch DH, Rodriguez N (2004). Heat shock protein 60 from Chlamydia pneumoniae elicits an unusual set of inflammatory responses via Toll-like receptor 2 and 4 in vivo.. Eur J Immunol.

[21] Bulut Y, Faure E, Thomas L, Karahashi H, Michelsen KS (2002). Chlamydial heat shock protein 60 activates macrophages and endothelial cells through Toll-like receptor 4 and MD2 in a MyD88-dependent pathway.. J Immunol.

[22] Costa CP, Kirschning CJ, Busch D, Dürr S, Jennen L (2002). Role of chlamydial heat shock protein 60 in the stimulation of innate immune cells by Chlamydia pneumoniae.. Eur J Immunol.

[23] Cao F, Castrillo A, Tontonoz P, Re F, Byrne GI (2007). Chlamydia pneumoniae—induced macrophage foam cell formation is mediated by Toll-like receptor 2.. Infect Immun.

[24] Kalayoglu MV, Byrne GI (1998). A Chlamydia pneumoniae component that induces macrophage foam cell formation is chlamydial lipopolysaccharide.. Infect Immun.

[25] Joyee AG, Yang X (2008). Role of toll-like receptors in immune responses to chlamydial infections.. Curr Pharm Des.

[26] Chen S, Sorrentino R, Shimada K, Doherty MT, Crother RT (2008). Chlamydia pneumoniae-induced foam cell formation requires MyD88 dependent and independent signaling and is reciprocally modulated by LXR activation.. J Immunol.

[27] Moulder JW (1991). Interaction of chlamydiae and host cells in vitro.. Mircobiol Rev.

[28] Wyrick PB (2000). Intracellular survival by Chlamydia.. Cell Microbiol.

[29] Heuer D, Lipinski AR, Machuy N, Karlas A, Wehrens A (2008). Chlamydia causes fragmentation of the Golgi compartment to ensure reproduction.. Nature.

[30] Cortes C, Rzomp KA, Tvinnereim A, Scidmore MA, Wizel B (2007). Chlamydia pneumoniae inclusion membrane protein Cpn0585 interacts with multiple Rab GTPases.. Infect Immun.

[31] Rothfuchs AG, Trumstedt C, Wigzell H, Rottenberg ME (2004). Intracellular bacterial infection-induced IFN-gamma is critically but not solely dependent on Toll-like receptor 4-myeloid differentiation factor 88-IFN-alpha beta-STAT1 signaling.. J Immunol.

[32] Chin AI, Dempsey PW, Bruhn K, Miller JF, Xu Y (2002). Involvement of receptor-interacting protein 2 in innate and adaptive immune responses.. Nature.

[33] Girardin SE, Boneca IG, Carneiro LA, Antignac A, Jéhanno M (2003). Nod1 detects a unique muropeptide from gram-negative bacterial peptidoglycan.. Science.

[34] Kobayashi K, Inohara N, Hernandez LD, Galán JE, Núñez G (2002). RICK/Rip2/CARDIAK mediates signalling for receptors of the innate and adaptive immune systems.. Nature.

[35] Meylan E, Tschopp J, Karin M (2006). Intracellular pattern recognition receptors in the host response.. Nature.

[36] Strober W, Murray PJ, Kitani A, Watanabe T (2006). Signalling pathways and molecular interactions of NOD1 and NOD2.. Nat Rev Immunol.

[37] Girardin SE, Boneca IG, Viala J, Chamaillard M, Labigne A (2003). Nod2 is a general sensor of peptidoglycan through muramyl dipeptide (MDP) detection.. J Biol Chem.

[38] Bertin J, Nir WJ, Fischer CM, Tayber OV, Errada PR (1999). Human CARD4 protein is a novel CED-4/Apaf-1 cell death family member that activates NF-kappaB.. J Biol Chem.

[39] Inohara N, Koseki T, del Peso L, Hu Y, Yee C (1999). Nod1, an Apaf-1-like activator of caspase-9 and nuclear factor-kappaB.. J Biol Chem.

[40] Inohara N, Koseki T, Lin J, del Peso L, Lucas PC (2000). An induced proximity model for NF-kappa B activation in the Nod1/RICK and RIP signaling pathways.. J Biol Chem.

[41] Yang Y, Yin C, Pandey A, Abbott D, Sassetti C (2007). Yang Y, Yin C, Pandey A, Abbott D, Sassetti C, Kelliher MA.. J Biol Chem.

[42] Park JH, Kim YG, McDonald C, Kanneganti TD, Hasegawa M (2007). RICK/RIP2 mediates innate immune responses induced through Nod1 and Nod2 but not TLRs.. J Immunol.

[43] Masumoto J, Yang K, Varambally S, Hasegawa M, Tomlins SA (2006). Nod1 acts as an intracellular receptor to stimulate chemokine production and neutrophil recruitment in vivo.. J Exp Med.

[44] Tada H, Aiba S, Shibata K, Ohteki T, Takada H (2005). Synergistic effect of Nod1 and Nod2 agonists with toll-like receptor agonists on human dendritic cells to generate interleukin-12 and T helper type 1 cells.. Infect Immun.

[45] Yang ZP, Cummings PK, Patton DL, Kuo CC (1994). Ultrastructural lung pathology of experimental Chlamydia pneumoniae pneumonitis in mice.. J Infect Dis.

[46] MacMicking J, Xie QW, Nathan C (1997). Nitric oxide and macrophage function.. Annu Rev Immunol.

[47] Opitz B, Förster S, Hocke AC, Maass M, Schmeck B (2005). Nod1-mediated endothelial cell activation by Chlamydophila pneumoniae.. Circ Res.

[48] Inohara N, Chamaillard M, McDonald C, Nuñez G (2005). NOD-LRR proteins: role in host-microbial interactions and inflammatory disease.. Annu Rev Biochem.

[49] Ojielo CI, Cooke K, Mancuso P, Standiford TJ, Olkiewicz KM (2003). Defective phagocytosis and clearance of Pseudomonas aeruginosa in the lung following bone marrow transplantation.. J Immunol.

[50] Gold ES, Simmons RM, Petersen TW, Campbell LA, Kuo CC (2004). Amphiphysin IIm is required for survival of Chlamydia pneumoniae in macrophages.. J Exp Med.

[51] McCully ML, Fairhead T, Colmont CS, Beasley FC, Heinrichs DE (2008). Receptor-Interacting Protein-2 Deficiency Delays Macrophage Migration and Increases Intracellular Infection during Peritoneal Dialysis-Associated Peritonitis.. Am J Nephrol.

[52] Kim JG, Lee SJ, Kagnoff MF (2004). Nod1 is an essential signal transducer in intestinal epithelial cells infected with bacteria that avoid recognition by toll-like receptors.. Infect Immun.

[53] Travassos LH, Carneiro LA, Girardin SE, Boneca IG, Lemos R (2005). Nod1 participates in the innate immune response to Pseudomonas aeruginosa.. J Biol Chem.

[54] Gandotra S, Jang S, Murray PJ, Salgame P, Ehrt S (2007). Nucleotide-binding oligomerization domain protein 2-deficient mice control infection with Mycobacterium tuberculosis.. Infect Immun.

[55] Viala J, Chaput C, Boneca IG, Cardona A, Girardin SE (2004). Nod1 responds to peptidoglycan delivered by the Helicobacter pylori cag pathogenicity island.. Nat Immunol.

[56] Welter-Stahl L, Ojcius DM, Viala J, Girardin S, Liu W (2006). Stimulation of the cytosolic receptor for peptidoglycan, Nod1, by infection with Chlamydia trachomatis or Chlamydia muridarum.. Cell Microbiol.

[57] Allen LA (2003). Mechanisms of pathogenesis: evasion of killing by polymorphonuclear leukocytes.. Microbes Infect.

[58] Laskay T, van Zandbergen G, Solbach W (2003). Neutrophil granulocytes—Trojan horses for Leishmania major and other intracellular microbes?. Trends Microbiol.

[59] Rodriguez N, Fend F, Jennen L, Schiemann M, Wantia N (2005). Polymorphonuclear neutrophils improve replication of Chlamydia pneumoniae in vivo upon MyD88-dependent attraction.. J Immunol.

[60] Rothfuchs AG, Kreuger MR, Wigzell H, Rottenberg ME (2004). Macrophages, CD4+ or CD8+ cells are each sufficient for protection against Chlamydia pneumoniae infection through their ability to secrete IFN-gamma.. J Immunol.

[61] Azenabor AA, Chaudhry AU (2003). Chlamydia pneumoniae survival in macrophages is regulated by free Ca2+ dependent reactive nitrogen and oxygen species.. J Infect.

[62] Frucht DM, Fukao T, Bogdan C, Schindler H, O'Shea JJ (2001). IFN-gamma production by antigen-presenting cells: mechanisms emerge.. Trends Immunol.

[63] Tötemeyer S, Sheppard M, Lloyd A, Roper D, Dowson C (2006). IFN-gamma enhances production of nitric oxide from macrophages via a mechanism that depends on nucleotide oligomerization domain-2.. J Immunol.

[64] Magalhaes JG, Philpott DJ, Nahori MA, Jéhanno M, Fritz J (2005). Murine Nod1 but not its human orthologue mediates innate immune detection of tracheal cytotoxin.. EMBO Rep.

[65] Buchholz KR, Stephens RS (2008). The cytosolic pattern recognition receptor NOD1 induces inflammatory interleukin-8 during Chlamydia trachomatis infection.. Infect Immun.

[66] Hatch T (1998). Chlamydia: old ideas crushed, new mysteries bared.. Science.

[67] Hesse L, Bostock J, Dementin S, Blanot D, Mengin-Lecreulx D (2003). Functional and biochemical analysis of Chlamydia trachomatis MurC, an enzyme displaying UDP-N-acetylmuramate:amino acid ligase activity.. J Bacteriol.

[68] McCoy AJ, Sandlin RC, Maurelli AT (2003). In vitro and in vivo functional activity of Chlamydia MurA, a UDP-N-acetylglucosamine enolpyruvyl transferase involved in peptidoglycan synthesis and fosfomycin resistance.. J Bacteriol.

[69] Chopra I, Storey C, Falla TJ, Pearce JH (1998). Antibiotics, peptidoglycan synthesis and genomics: the chlamydial anomaly revisited.. Microbiology.

[70] Fox A, Rogers JC, Gilbart J, Morgan S, Davis CH (1990). Muramic acid is not detectable in Chlamydia psittaci or Chlamydia trachomatis by gas chromatography-mass spectrometry.. Infect Immun.

[71] McCoy AJ, Adams NE, Hudson AO, Gilvarg C, Leustek T (2006). L,L-diaminopimelate aminotransferase, a trans-kingdom enzyme shared by Chlamydia and plants for synthesis of diaminopimelate/lysine.. Proc Natl Acad Sci U S A.

[72] McCoy AJ, Maurelli AT (2006). Building the invisible wall: updating the chlamydial peptidoglycan anomaly.. Trends Microbiol.

[73] Hsia RC, Pannekoek Y, Ingerowski E, Bavoil PM (1997). Type III secretion genes identify a putative virulence locus of Chlamydia.. Mol Microbiol.

[74] Cox RL, Kuo CC, Grayston JT, Campbell LA (1988). Deoxyribonucleic acid relatedness of Chlamydia sp. strain TWAR to Chlamydia trachomatis and Chlamydia psittaci.. International Journal of Systematic Bacteriology.

[75] Peterson EM, de la Maza LM, Brade L, Brade H (1999). Characterization of a neutralizing monoclonal antibody directed at the lipopolysaccharide of Chlamydia pneumoniae.. Infect Immun.

