# Correction: The NOD/RIP2 Pathway Is Essential for Host Defenses Against *Chlamydophila pneumoniae* Lung Infection

**DOI:** 10.1371/annotation/0f4b8a96-1598-4c90-b816-8fbe7614310b

**Published:** 2009-04-14

**Authors:** Kenichi Shimada, Shuang Chen, Paul W. Dempsey, Rosalinda Sorrentino, Randa Alsabeh, Anatoly V. Slepenkin, Ellena Peterson, Terence M. Doherty, David Underhill, Timothy R. Crother, Moshe Arditi

There were two errors in the second sentence in the legend to Figure S3. The correct text should read: BMDMs were grown and isolated from WT or Rip2^-/-^ mice and adoptively transferred intratracheally into the airways of WT mice or Rip2^-/-^ mice (5x10^5^/mouse) together with C. pneumoniae (1x10^6^ IFU).

