# Correction: The NOD/RIP2 Pathway Is Essential for Host Defenses Against *Chlamydophila pneumoniae* Lung Infection

**DOI:** 10.1371/annotation/f3aa682e-3bc2-4a05-ac7f-05c6cfe1bbd7

**Published:** 2009-04-14

**Authors:** Kenichi Shimada, Shuang Chen, Paul W. Dempsey, Rosalinda Sorrentino, Randa Alsabeh, Anatoly V. Slepenkin, Ellena Peterson, Terence M. Doherty, David Underhill, Timothy R. Crother, Moshe Arditi

There was an error in the last sentence of the legend to Figure 4.

The correct text should read: Representative histograms were shown to analyze relative bacterial number in macrophages (MAC) and neutrophils (Neu) between WT (gray-filled histogram) and Rip2^-/-^ (thin line histogram).

