# Correction: The NOD/RIP2 Pathway Is Essential for Host Defenses Against *Chlamydophila pneumoniae* Lung Infection

**DOI:** 10.1371/annotation/289c756f-6d61-4872-83d8-4bd5a1b4f226

**Published:** 2009-04-14

**Authors:** Kenichi Shimada, Shuang Chen, Paul W. Dempsey, Rosalinda Sorrentino, Randa Alsabeh, Anatoly V. Slepenkin, Ellena Peterson, Terence M. Doherty, David Underhill, Timothy R. Crother, Moshe Arditi

There were errors in the second and fourth sentences of the legend to Figure S1. In each instance, in what appears as (2x105), the 5 should be superscripted. e.g., (2x10^5^).

