# Supplementary figures and images for: The NOD/RIP2 Pathway Is Essential for Host Defenses Against Chlamydophila pneumoniae Lung Infection
Source: PLoS Pathog. 2009 Apr 10;5(4):e1000379. doi: 10.1371/journal.ppat.1000379 (PMC2660273; doi:10.1371/journal.ppat.1000379)

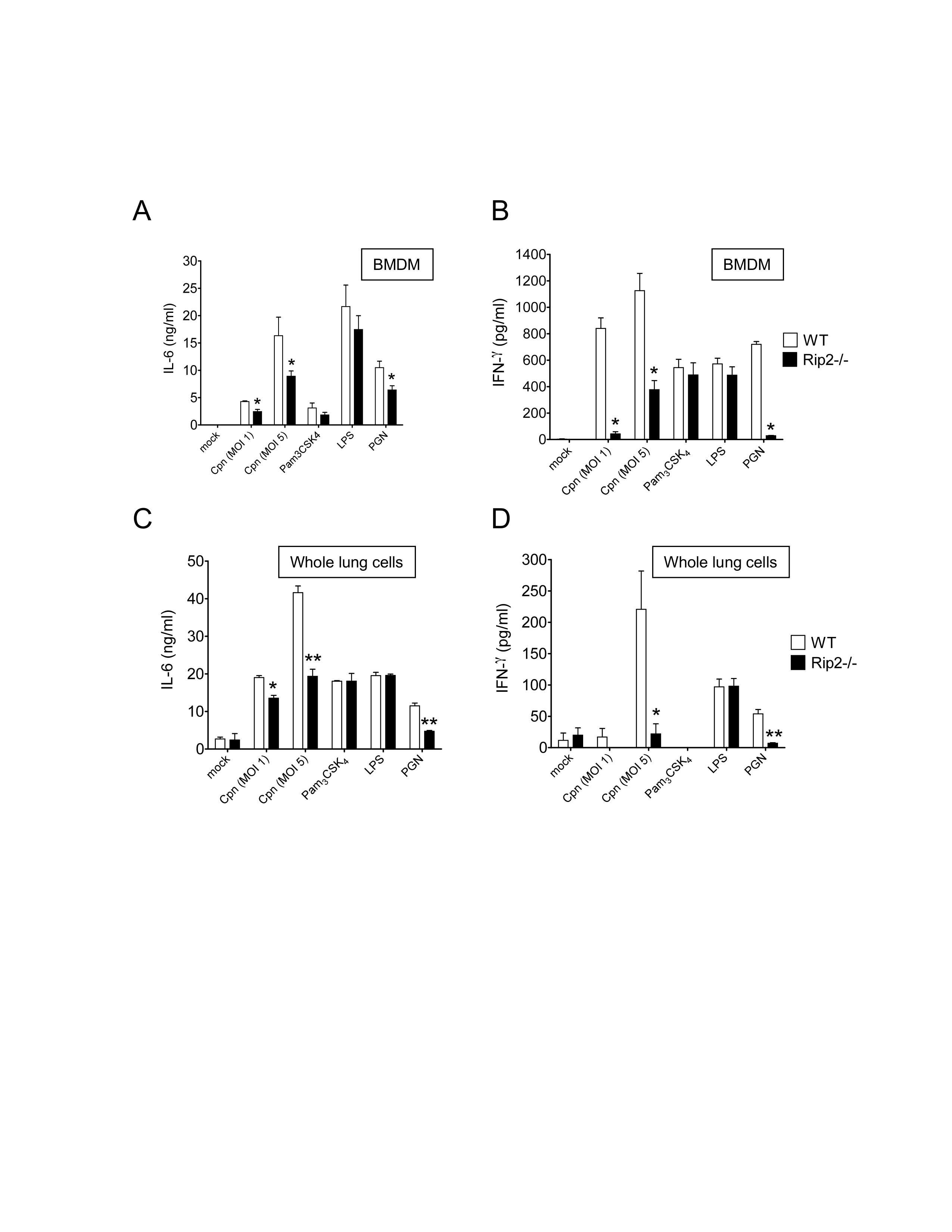

Supplement: Figure S1 — IL-6 and IFN-γ production in Rip2−/− BMDM and whole lung cells. BMDM (2×10^5^ cells) were stimulated with C. pneumoniae (Cpn) for 24 hours. IL-6 (A) and IFN-γ (B) concentration in the supernatant was evaluated by ELISA. Whole lung cells (2×10^5^ cells) were stimulated with C. pneumonia (Cpn) for 24 hours. IL-6 (C) and IFN-γ (D) concentration in the supernatant was evaluated by ELISA. Data shown are representative of three independent experiments. Statistical significance was determined by Student's t test (*p<0.05, **p<0.01, n = 3). (0.32 MB TIF) [file ppat.1000379.s001.tif]

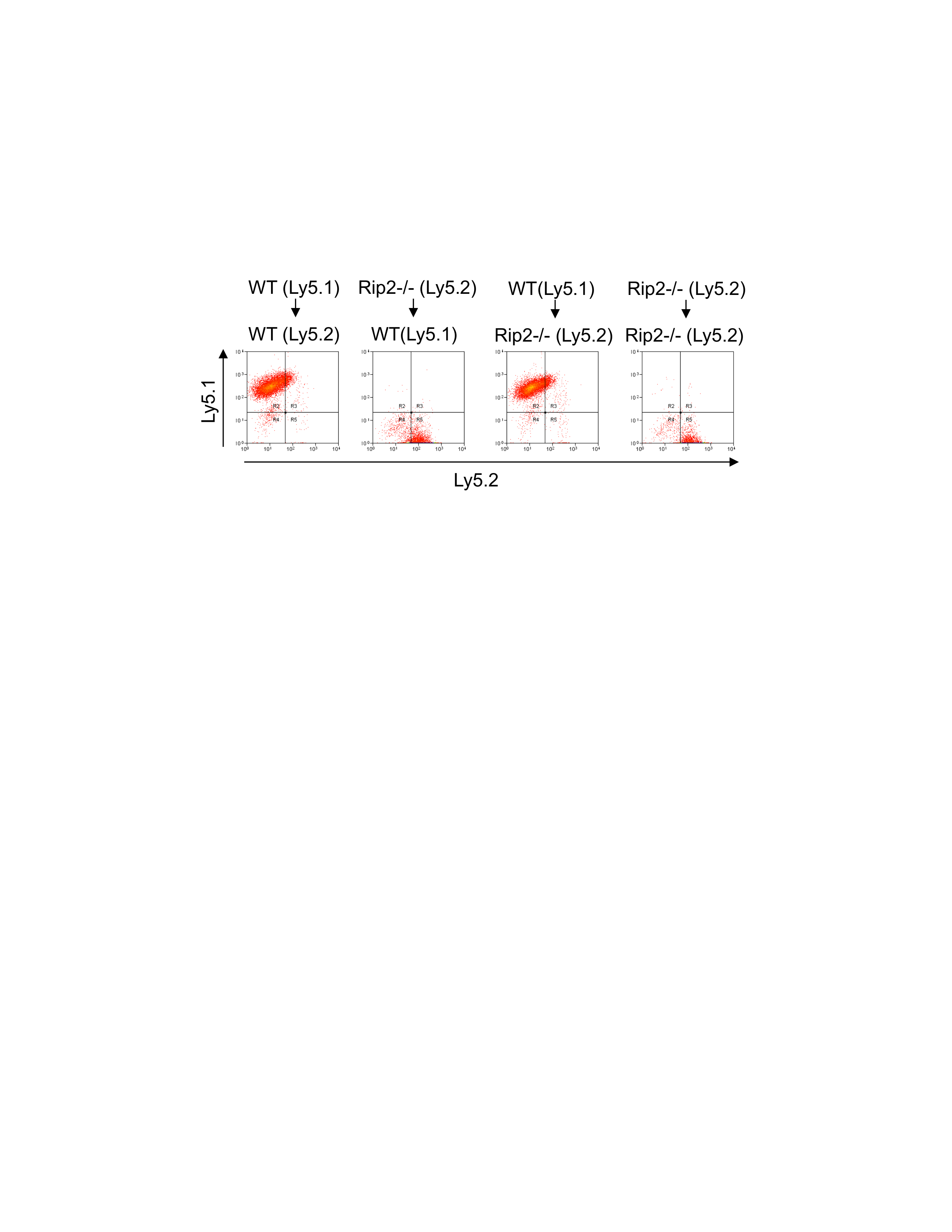

Supplement: Figure S2 — Reconstitution of BM chimeras. Recipient mice were lethally irradiated and reconstituted with BM from WT or Rip2−/− donor mice. After 7 weeks, lungs were removed, digested with collagenase and DNase I. Cells were stained with FITC-anti-Ly5.2 mAB and PE-anti-Ly5.1 mAb and analyzed by FACScan. (0.25 MB TIF) [file ppat.1000379.s002.tif]

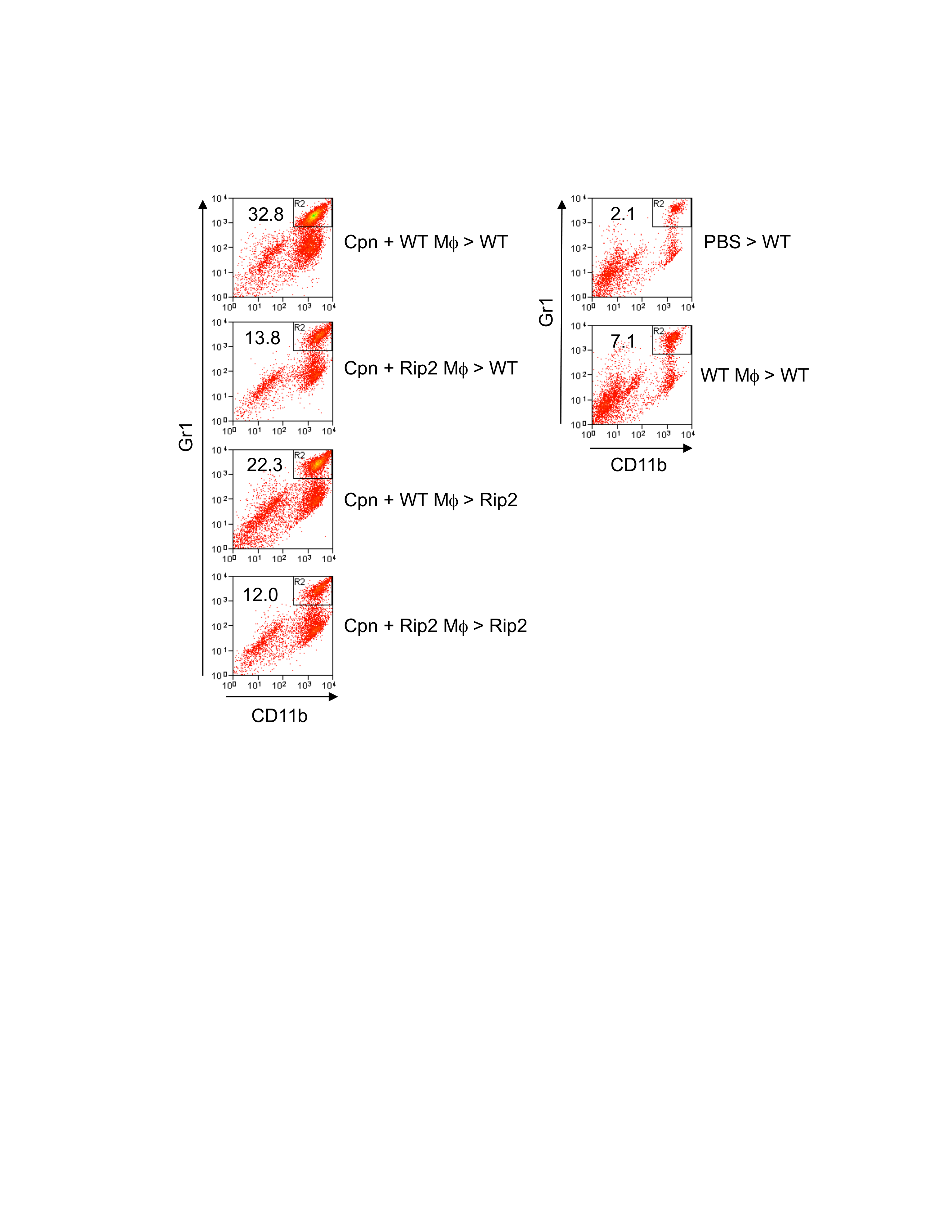

Supplement: Figure S3 — Neutrophil recruitment after WT BMDMs were adoptively transferred into Rip2−/− mice. BMDMs were grown and isolated from WT or Rip2−/− mice and adoptively transferred intratracheally into the airways of WT mice or Rip2−/− mice (5×10^5^/mouse) together with C. pneumoniae (1×10^6^ IFU). BMDMs were first incubated with C. pneumoniae in vitro for 30 minutes prior to intratracheal administration. After 3 days, lungs were collected and cells were stained with PE-anti-GR1 mAB and PECy5-anti-CD11b mAb and analyzed by FACScan. The percentages of gated positive cells are indicated. Data shown are representative of two independent experiments. (0.64 MB TIF) [file ppat.1000379.s003.tif]

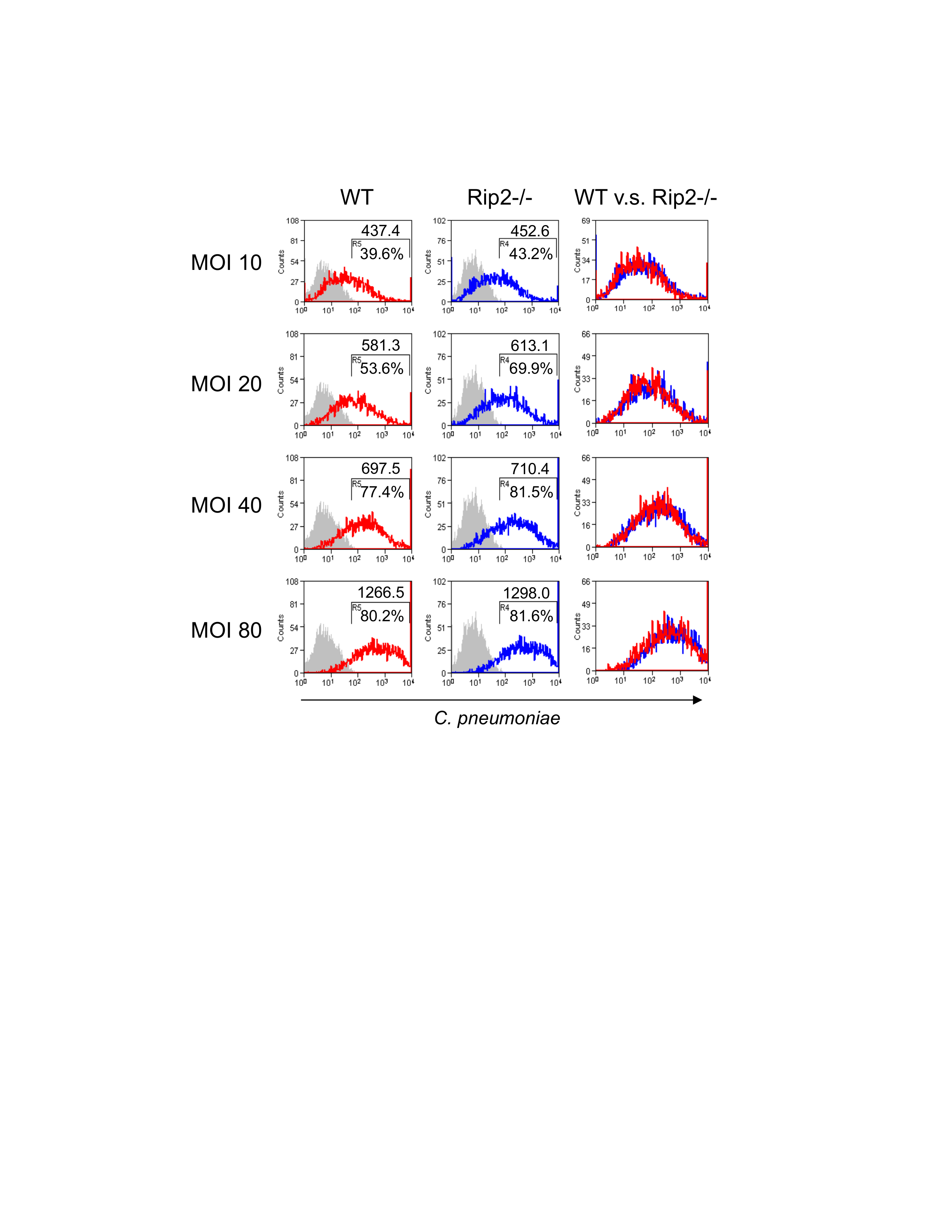

Supplement: Figure S4 — Phagocytosis of labeled C. pneumoniae by BMDM is unaffected by targeted deletion of the gene for Rip2. The bacteria were incubated with DyLigntTM 633 NHS-Ester reagent (Thermo Scientific, Rockford, IL, USA) for 1 hour at RT. FBS was added to stop the reaction, washed with PBS, and centrifuged at 18,000 rpm (60,000× g) for 1 hour. The supernatant was carefully aspirated and the bacterial pellet was resuspended in cell culture medium. BMDMs were exposed to labelled C. pneumoniae (solid line histogram) or vehicle control (gray-filled histogram) by centrifugation at 500× g for 30 minutes at 4°C, then incubated for 2 hours at 37°C. MOIs of 10, 20, 40, and 80 were used. Uninternalized bacteria were removed by incubating the cells in Trypsin/EDTA for 10 minutes at 37°C as previously described [75]. The cells were washed and fixed with 2% formalin/PBS, and analyzed by FACS. The mean fluorescence intensity (MFI) and percentage of labeled C. pneumoniae internalized cells were indicated. Data are representative of two independent experiments. (0.50 MB TIF) [file ppat.1000379.s004.tif]

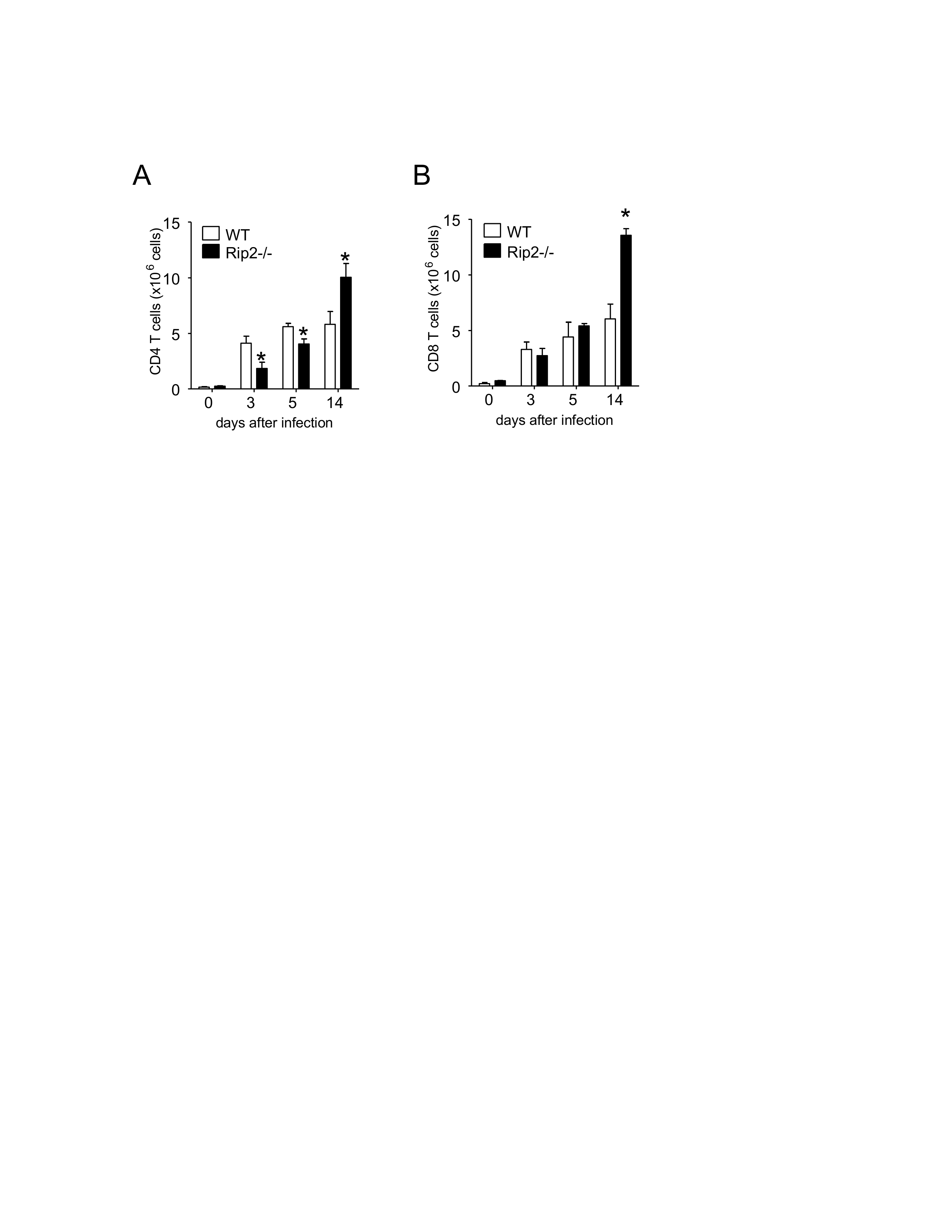

Supplement: Figure S5 — Delayed CD4 T cell recruitment to the lungs in Rip2−/− mice. C. pneumoniae was inoculated intratracheally into Rip2−/− and WT mice (1×1 06 IFU/mouse). Lungs were removed on days 0, 3, 5, and 14 post-infection, digested with collagenase and DNase I. Cells were stained with FITC-anit-CD4 mAb, PE-anti-CD3 mAB, and PECy5-anti-CD8 mAb, and analyzed by FACScan. The total number of CD4+CD3+ (A) and CD8+CD3+ (B) T cells in lungs were counted. Data shown are representative of four independent experiments. Statistical significance was determined by Student's t test (*p<0.05). (0.15 MB TIF) [file ppat.1000379.s005.tif]
